# Olanzapine Overdose in a Two-Year-Old Girl Resulting in Both High Serum and Plasma Levels

**DOI:** 10.7759/cureus.43002

**Published:** 2023-08-05

**Authors:** Koji Yokoyama, Toshinari Yakuo, Mitsukazu Mamada, Masashi Nagata

**Affiliations:** 1 Department of Pediatrics, Japanese Red Cross Wakayama Medical Center, Wakayama, JPN; 2 Department of Pharmacy, Tokyo Medical and Dental University Hospital, Tokyo, JPN

**Keywords:** impaired consciousness, proper specimen storage, blood concentration measurement, toxicity, olanzapine

## Abstract

The antipsychotic olanzapine is used increasingly to treat various psychiatric illnesses. Accidental olanzapine overdose is uncommon among children. Here, we report a case of a child presenting with an unexplained coma. Accidental ingestion of olanzapine (20 mg) was confirmed by measurement of drug concentrations in both serum and plasma.

## Introduction

The antipsychotic olanzapine is used increasingly to treat various psychiatric illnesses [1]. Accidental olanzapine overdose with confirmation by blood concentration measurement is uncommon among children [2,3]. Here, we report a 31-month-old girl presenting with altered consciousness caused by accidental consumption of olanzapine (20 mg); the overdose was confirmed by measuring both serum and plasma concentrations. Thus, serum and plasma samples can be used to check for acute olanzapine toxicity in children. Measurement of drug concentrations in the blood revealed the cause of the unexplained coma in this patient. In pediatric cases with unexplained loss of consciousness, we should suspect accidental drug ingestion and obtain and test appropriate specimens.

## Case presentation

**Figure 1:**
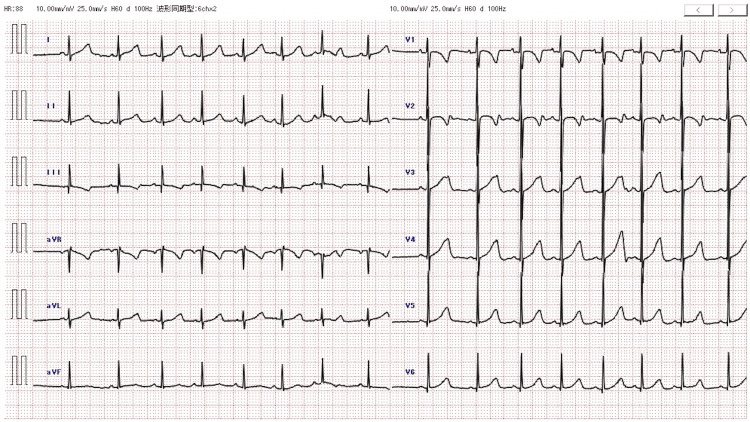
Electrocardiogram at the emergency department visit showed normal intervals

**Figure 2:**
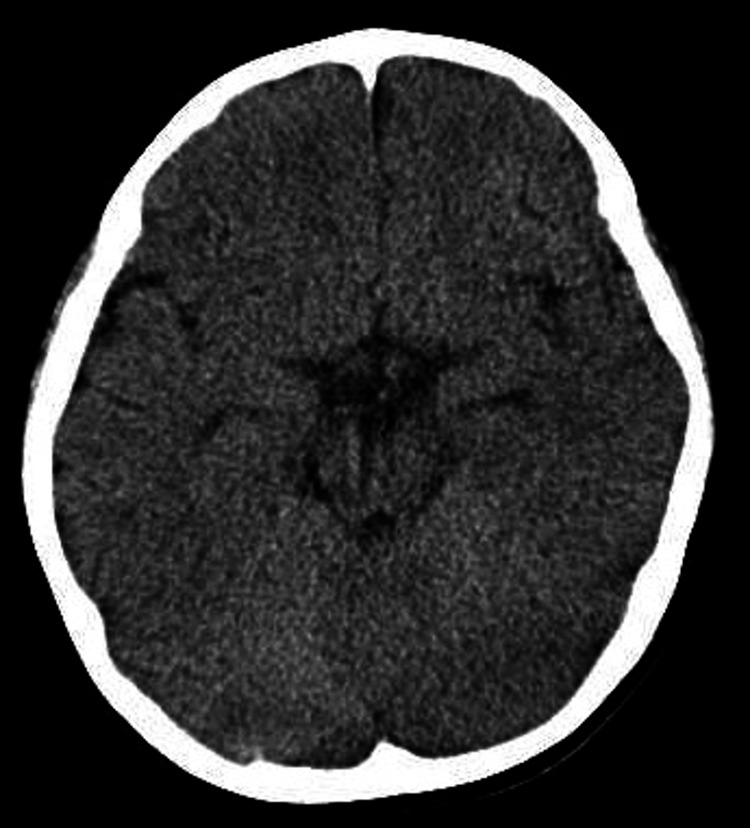
Brain computed tomography at the emergency department visit revealed no abnormality

A 31-month-old, previously healthy girl weighing 14.4 kg, was brought to our hospital due to a 90-minute period of somnolence. On admission, she was sleeping (snoring) and did not open her eyes after a painful stimulus. Her vital signs were as follows: her blood pressure was 75/58 mmHg, her pulse was 90 beats/minute, her respiratory rate was 30 breaths/minute, her tympanic temperature was 36.6°C, and her oxygen saturation was 100% on room air. Her Glasgow Coma Scale score was 3 (eye-opening was 1 point, verbal response was 1 point, and motor response was 1 point). She had constricted pupils, with a normal light reflex. Clinical laboratory, electrocardiogram, and brain computed tomography analyses revealed no abnormalities (Figure 1 and Figure 2). At first, the cause of unconsciousness was unknown. She was hospitalized for close observation and started on maintenance fluids. Later, her parents reported that two 10 mg olanzapine tablets were missing at home. After 30 hours, she had recovered fully, with normal levels of consciousness.

Olanzapine intoxication was confirmed by measurement of serum and plasma olanzapine levels (144 ng/mL and 151 ng/mL, respectively; therapeutic serum level in adults is approximately 5-75 ng/mL and plasma level is 20-50 ng/mL, respectively) [4,5]. Serum and plasma levels measured at 14 days post-ingestion were 0 ng/mL. Olanzapine concentrations were measured by modified high-performance liquid chromatography, as described previously [6].

## Discussion

The clinical course of this patient provides two important clinical indications. First, both serum and plasma samples can be used to check for acute olanzapine toxicity in an infant. In pediatric practice, it is often difficult to obtain enough specimens at the site of emergency medical care [7,8]. Also, to enable testing and analysis, appropriate specimen storage is important [9,10]. Here, we tested both plasma and serum samples. Numerous studies have investigated the association between serum/plasma concentrations of olanzapine and metabolic abnormalities [11,12]; however, it is not clear which type of sample is better for accurate measurement of olanzapine concentrations in blood. The pharmacokinetic parameters of olanzapine in adults suggest that blood levels correlate linearly with dose, with 60% of the drug being bioavailable and approximately 93% bound to protein [13]. One notable advantage of serum-based measurement is the simplicity of collection and storage. In addition, the serum is commonly used for diagnostic tests, making it a familiar and standardized choice. The major drawback is the absence of clotting factors, which may interfere with coagulation or hemolysis and result in erroneous measurement of biomarkers. Plasma is a richer source of blood components such as blood cells, clotting factors, and various proteins, making it a suitable choice for a wide range of tests. The main disadvantage of plasma is that it requires specialized collection and storage [9,14,15]. Second, blood concentration measurements identified the cause of the unexplained unconsciousness in this case. There are many reasons for the loss of consciousness in children. Indeed, intoxication is the cause of non-traumatic coma in 10.3% of pediatric cases in England [16]. It is difficult to identify the cause from clinical findings; a careful interview with the guardian is important for the identification of the causative agent [17]. But in the current case, drug ingestion was not witnessed; therefore, we could not confirm the dose or time of ingestion. Measurement of drug levels in blood enabled us to identify the cause of unconsciousness in this case.

## Conclusions

Analysis of both serum and plasma samples identified the cause of unexplained pediatric unconsciousness as olanzapine toxicity. Further data are required to determine whether appropriate specimen storage methods should be introduced in clinical situations and whether the measurement of drug concentrations in blood reliably identifies the cause of unexplained coma.
